# HEALTH PROFILE OF PORTUGUESE CENTENARIANS FROM 2011 TO 2021: AN UPDATE BASED ON NATIONAL CENSUS

**DOI:** 10.1093/geroni/igad104.0917

**Published:** 2023-12-21

**Authors:** Oscar Ribeiro, Lia Araujo, Constanca Paul, Laetitia Teixeira

**Affiliations:** University of Aveiro, Aveiro, Aveiro, Portugal; Cintesis, Porto, Porto, Portugal; ICBAS, Portugal, Porto, Porto, Portugal; School of Medicine and Biomedical Sciences, University of Porto, Porto, Porto, Portugal

## Abstract

According to the Portuguese National Census, the number of centenarians increased from 1526 (82.1% women) in 2011 to 2801 (82.0% women) in 2021. Along with a description of main sociodemographic characteristics, this paper provides an updated health profile of this population based on their perceived difficulties in six functional domains of basic actions (seeing, hearing, walking, cognition, self-care, and communication) as assessed by a set of questions developed by the Washington Group on Disability Statistics that is consistent with the International Classification of Functioning, Disability, and Health (ICF). Sex differences are also presented, as well as comparisons between data from Census 2011 and Census 2021. Most centenarians show major constraints in their mobility and personal care (76.3% cannot/have great difficulties in walking/climbing stairs, and 72.8% cannot/have great difficulties in bathing/dressing), and a better outcome is reported in communication (64.2% report no/reduced difficulty for understanding/being understood). This profile globally follows the pattern observed in 2011, but with fewer centenarians reporting such level of difficulties. The percentage of centenarians reporting no/reduced difficulties in seeing and hearing increased from 32.6% and 27.7% in 2011, to 54.4% and 44.8% in 2021, respectively. In both censuses, women presented a higher percentage of difficulties in all health-related variables. Globally, this comparative perspective shows improvements in the perceived health profile of the later born cohort of Portuguese centenarians and features the continued need for addressing gender-specific and gender-sensitive interventions that acknowledge older women’s care needs.

